# XOR inhibition with febuxostat accelerates pulmonary endothelial barrier recovery and improves survival in lipopolysaccharide‐induced murine sepsis

**DOI:** 10.14814/phy2.13377

**Published:** 2017-08-11

**Authors:** Mahendra Damarla, Laura F. Johnston, Gigi Liu, Li Gao, Lan Wang, Lidenys Varela, Todd M. Kolb, Bo S. Kim, Rachel L. Damico, Paul M. Hassoun

**Affiliations:** ^1^ Department of Medicine Johns Hopkins University School of Medicine Baltimore Maryland

**Keywords:** Febuxostat, lipopolysaccharide, mortality, organ dysfunction, oxidative damage, sepsis, xanthine oxidoreductase

## Abstract

Sepsis is a leading cause of death among patients in the intensive care unit, resulting from multi‐organ failure. Activity of xanthine oxidoreductase (XOR), a reactive oxygen species (ROS) producing enzyme, is known to be elevated in nonsurvivors of sepsis compared to survivors. We have previously demonstrated that XOR is critical for ventilator‐induced lung injury. Using febuxostat, a novel nonpurine inhibitor of XOR, we sought to determine the role of XOR inhibition in a murine model of sepsis‐induced lung injury and mortality. C57BL/6J mice were subjected to intravenous (IV) lipopolysaccharide (LPS) for various time points, and lungs were harvested for analyses. Subsets of mice were treated with febuxostat, pre or post LPS exposure, or vehicle. Separate groups of mice were followed up for mortality after LPS exposure. After 24 hr of IV LPS_,_ mice exhibited an increase in XOR activity in lung tissue and a significant increase in pulmonary endothelial barrier disruption. Pretreatment of animals with febuxostat before exposure to LPS, or treatment 4 h after LPS, resulted in complete abrogation of XOR activity. Inhibition of XOR with febuxostat did not prevent LPS‐induced pulmonary vascular permeability at 24 h, however, it accelerated recovery of the pulmonary endothelial barrier integrity in response to LPS exposure. Furthermore, treatment with febuxostat resulted in significant reduction in mortality. Inhibition of XOR with febuxostat accelerates recovery of the pulmonary endothelial barrier and prevents LPS‐induced mortality, whether given before or after exposure to LPS.

## Introduction

Sepsis is a devastating illness with an annual incidence of 750,000, representing nearly 10% of all intensive care unit (ICU) admissions with a mortality approaching 30% (Angus and van der Poll 2013). Sepsis constitutes a complex systemic inflammatory response initiated by microbial factors that perpetuate proinflammatory mediators, a key pathophysiological consequence of sepsis leading to endothelial barrier disruption (Angus and van der Poll 2013; Goldenberg et al. 2011). Injury to this barrier in any given vital organ leads to extravasation of proteinaceous fluid as well as inflammatory cells from the intravascular space resulting in local tissue hypoxia, organ dysfunction, and ultimately organ failure (Angus and van der Poll 2013; Goldenberg et al. 2011). Probability of mortality from sepsis increases with each additional failed organ (Mayr et al. 2014). Currently, therapy relies heavily on early recognition of sepsis with prompt initiation of antimicrobial agents and supportive care (Angus and van der Poll 2013; Dellinger et al. 2013).

Pathophysiological mechanisms of organ dysfunction observed in sepsis are thought to be related to an exuberant inflammatory state which results in collateral tissue damage (Goldenberg et al. 2011). Specifically, reactive oxygen species (ROS) play a key pathogenic role in sepsis‐induced organ dysfunction (Boueiz et al. 2008; Fink 2002). Recent human studies have shown that oxidative damage is a prominent feature in sepsis‐induced organ failure (Galley et al. 1996; Luchtemberg et al. 2008; Rice et al. 2011; Ware et al. 2011) stressing a role for attenuating oxidative damage as a mechanism of treating sepsis.

Because of its ability to generate ROS, xanthine oxidoreductase (XOR) plays an essential role in the pathogenesis of various organ dysfunctions (Boueiz et al. 2008). Interestingly, XOR activity is elevated in patients with sepsis when compared with healthy volunteers and noninfected patients (Galley et al. 1996). A role for oxidant injury in sepsis‐induced organ dysfunction has been studied extensively in animal models (Fink 2002). Our group has demonstrated inhibition of XOR with allopurinol is protective in rodent models of acute lung injury, ventilator‐induced lung injury, and cigarette smoke lung injury (Abdulnour et al. 2006; Hassoun et al. 1998; Kim et al. 2013; Le et al. 2008). With the recent advent of a specific XOR inhibitor, febuxostat, additional studies investigating the role of this enzyme in many disease states are emerging (Lee et al. 2014; Nomura et al. 2014).

To that end, we hypothesized that, in a murine model of sepsis, XOR mediated oxidative damage leads to worsened lung injury, and inhibiting XOR with febuxostat would protect against endotoxemia‐induced lung injury.

## Materials and Methods

The Johns Hopkins University Institutional Animal Care and Use Committee approved all animal protocols.

### Experimental protocol and animal exposure to intravenous LPS

Male C57BL/6J (wild type, WT) mice aged 10–12 weeks (Jackson Laboratory, Bar Harbor, ME) were randomly exposed to intravenous PBS or lipopolysaccharide (LPS, 0127:B8, Sigma) via retro‐orbital injection to mimic sepsis, as previously described (Yardeni et al. 2011). For survival studies, mice were weighed daily and followed until death or recovery. Food and water were available ad libitum.

### Febuxostat treatment

Febuxostat was obtained from Takeda Pharmaceuticals (Deerfield, IL). Febuxostat was crushed and dissolved in water and given via oral gavage at a dose of 5 mg/kg per day. For pretreatment with febuxostat, the first dose was administered the previous evening (~16 h before) and 30 min prior to LPS exposure. The treatment dosing of febuxostat started 4 h after LPS exposure. The febuxostat dosing was chosen based on previous publications (Kim et al. 2013). For longer time points, febuxostat was given once daily for the duration of the experiment.

### Assessment of lung injury

After exposure to experimental conditions, mice were sacrificed at the indicated time points. At the time of harvest, the main pulmonary artery was cannulated and the lungs were flushed free of blood with 2 mL of phosphate buffered saline. Pulmonary edema was assessed by determining the ratio of right lung wet weight to total body weight or the lung wet‐to‐dry weight ratios, both as described previously (Aggarwal et al. 2010; Damarla et al. 2014). Bronchoalveolar lavage fluid (BALF) was collected for assessment of cell counts and protein concentration, as previously described (Damarla et al. 2014; Singer et al. 2016).

### Biochemical studies of lung tissue

Lung tissues for biochemical assays were weighed at the time of harvest and then immediately snap‐frozen in liquid nitrogen for subsequent analysis. Enzymatic XOR activity of lung tissue homogenates was assessed using a fluorometric assay as previously described (Kayyali et al., 2001,2003; Kim et al. 2013). In short, xanthine oxidase activity is determined as the rate of oxidation of pterin to the fluorescent product isoxanthopterin. After which, methylene blue is added as an electron acceptor to measure the combined activities of both xanthine oxidase and xanthine dehydrogenase. The use of methylene blue obviates any fluorescence overlap that may be present with NADH and isoxanthopterin. The reaction is then inhibited by the addition of allopurinol. Lastly, the addition of a known concentration of isoxanthopterin serves as an internal standard for fluorescence quenching. The units of this assay are *μ*mol·min^−1^ g·tissue^−1^. We further adjust XOR activity for protein concentration. Lung myeloperoxidase activity was measured using an assay based on the colorimetric conversion of the substrate o‐dianisidine dihydrochloride as previously described (Yu et al. 2006). Lung tissue Resolvin E1 was assessed using an ELISA kit (MyBioSource; San Diego, CA), according to manufacturer's guidelines.

Immunoblotting of lung homogenates was performed using standard techniques as previously described (Kim et al. 2013). Antibodies directed at phospho‐Histone H2A.X (Ser139), *β*‐actin, Caspase 3 (Cell Signaling; Boston, MA), and CMKLR1 (Cayman Chemical, Ann Arbor, MI) were used. Quantification of immunoreactive bands was performed using ImageJ (Bethesda, MD).

### Statistics

Data are shown as mean ± SEM. We performed multiple comparisons by a nonparametric analysis of variance using the Kruskal–Wallis test. Comparison between two groups was performed using the nonparametric Mann–Whitney test. Nonparametric tests were used to not assume normal distribution of data. Survival curves were analyzed using the Log‐rank test. A *P* < 0.05 was considered significant. Data were analyzed using GraphPad Prism 7.

## Results

### Intravenous lipopolysaccharide induces XOR activity

**Figure 1:**
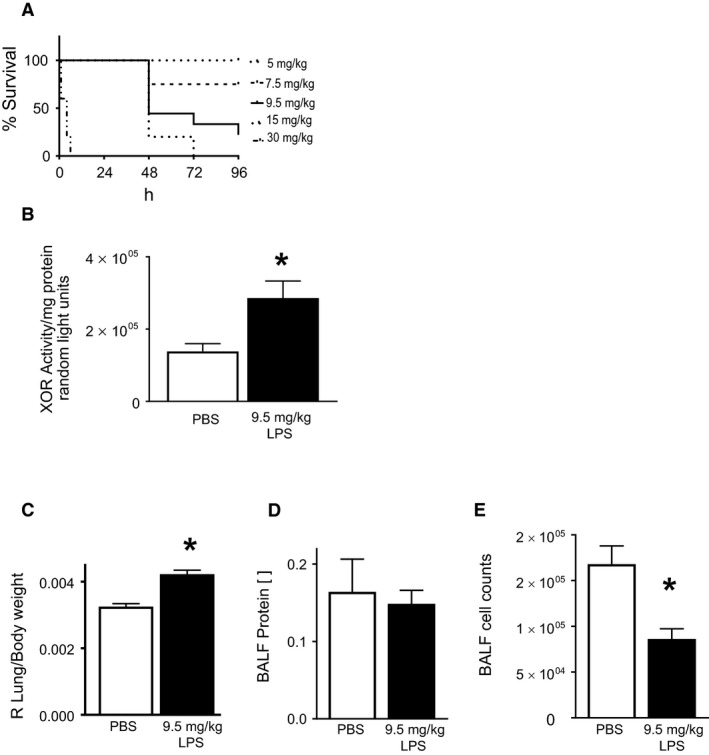
Intravenous exposure to lipopolysaccharide induces mortality and lung XOR activation in mice. (A) C57BL/6J mice were exposed to increasing concentrations of IV LPS and followed up for mortality. There is a dose‐dependent increase in mortality, with no mortality observed at 5 mg/kg of LPS. At 9.5 mg/kg of LPS there is no early mortality (day 1) but this increases over time. (B) WT mice were exposed to 9.5 mg/kg of LPS for 24 h after which lungs were harvested for XOR activity. After LPS exposure, there is a significant increase in XOR activation. (C) After 24 h of LPS exposure, there is a marked increase in pulmonary vascular permeability as measured by R lung‐wet weight to body weight ratio. (D) After 24 h of LPS exposure, there is no difference in protein concentrations from bronchoalveolar lavage fluid. (E) After 24 h of LPS exposure, there is a decrease in total cell counts in the bronchoalveolar lavage fluid. *N* = 4–13 mice per group. *P <* *0.05 versus all others. ^#^ *P *<* *0.05 versus LPS alone and control.

As sepsis leads to multi‐organ failure and eventual death, we sought to create a murine model of sepsis that mimics organ dysfunction and increased mortality. Adult male mice were subjected to intravenous LPS at increasing doses and followed up for assessment of mortality. As shown in Figure 1A, intravenous administration of LPS causes a dose‐dependent increase in mortality. A dose of 30 mg/kg of LPS leads to mortality within 6 h of LPS administration suggesting distributive shock followed by vascular collapse as the mechanism of death. Whereas, 5 mg/kg of LPS had no observed mortality, and was, therefore, considered a sublethal dose.

As we sought to create a severe sepsis model that results in lung injury, we chose a dose of 9.5 mg/kg of LPS for subsequent studies. As shown in Figure 1A, with 9.5 mg/kg of LPS there is no early, that is, within 24 h, mortality observed. At 24 h there is ~200% increase in XOR activity in lung tissue homogenates from mice exposed to LPS as compared to vehicle, Figure 1B. After 24 h of LPS exposure, there is a significant increase in pulmonary vascular permeability, as evidenced by an increase in the right lung wet weight to body weight ratio, Figure 1C. Further, exposure to IV LPS seemed to only affect the endothelial barrier as there is no evidence of an alveolitis (epithelial barrier injury), as evidenced by a similar BAL fluid protein concentration, Figure 1D, and a slightly decreased BAL fluid total cell count (Fig. 1E), similar to our previously published results (Damarla et al. 2014; Singer et al. 2016). These data implicate XOR (and potentially XOR‐induced ROS) as a potential determinant of lung injury.

### Inhibiting XOR with febuxostat does not improve lipopolysaccharide‐induced lung injury

**Figure 2:**
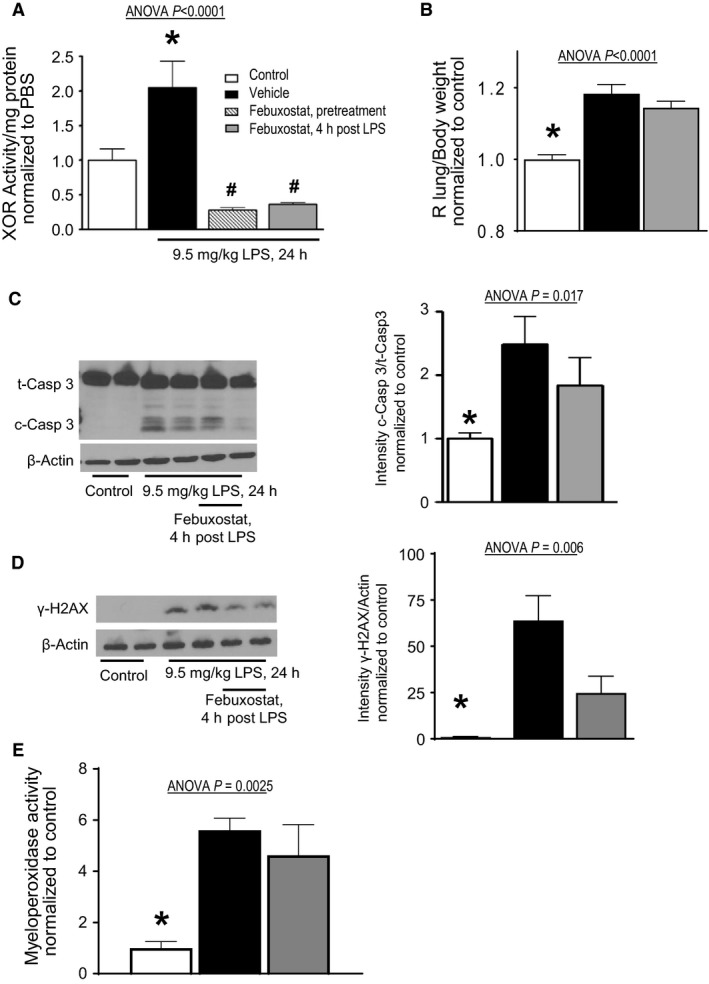
Inhibiting XOR with febuxostat does not prevent lipopolysaccharide‐induced lung injury. Mice were exposed to LPS and a subset were pretreated or therapeutically treated with febuxostat. Twenty‐four hours later lung tissue was harvested for analyses. (A) Therapeutic administration of febuxostat 4 h after LPS exposure inhibits day 1 LPS‐induced lung XOR activity similarly to pretreatment with febuxostat. (B) Pulmonary vascular permeability as measured by lung‐wet weight to body weight ratios was significantly higher in all mice exposed to LPS. This was not attenuated by febuxostat treatment. (C) Left Panel) A representative immunoblot reveals increased cleaved‐caspase 3, a marker of apoptosis, in response to LPS. Febuxostat treatment did not significantly attenuate caspase 3 cleavage; confirmed by densitometry (Right Panel). (D) Left Panel) A representative immunoblot reveals increased *γ*‐H2A.X, a marker of oxidative damage, in response to LPS. Febuxostat treatment did not significantly attenuate oxidative damage; confirmed by densitometry (Right Panel). (E) Neutrophil‐derived oxidants, as assessed by lung myeloperoxidase activity, was significantly higher in all mice exposed to LPS. Febuxostat treatment did not attenuate lung myeloperoxidase activity. *N* = 4–28 mice per group *, *P *<* *0.05 versus all others.

Many preclinical animal model studies of drug interventions have utilized a preventative strategy to test the efficacy of a particular therapeutic intervention where the drug is given prior to exposure to the injurious agent (Bernard et al. 1984; Xiang et al. 2003). In order to test the role of therapeutic inhibition of XOR, that is, after the initiation of injury, we first established a time course of XOR activation after exposure to LPS. There was a time‐dependent increase in XOR activity starting at 4 h after LPS exposure (data not shown). Four hours after LPS exposure was chosen for the timing of therapeutic XOR inhibition. As shown in Figure 2A, treatment with febuxostat completely abrogated XOR activity, whether given prior to LPS exposure (pretreatment) or 4 h after LPS exposure (therapeutic strategy).

Sepsis is a common etiology of the acute respiratory distress syndrome (ARDS) (Matthay et al. 2012). Additionally, oxidative damage has long been thought to play a pathogenic role in the development of ARDS (Bernard et al. 1984; Matthay et al. 2012). Further, we have previously demonstrated that inhibiting XOR with allopurinol is protective against murine ventilator‐induced lung injury (Abdulnour et al. 2006; Le et al. 2008). Therefore, we sought to determine if febuxostat, a more specific XOR inhibitor (Becker et al. 2005; Edwards 2009; Ernst and Fravel 2009), could protect against endotoxemia‐induced lung injury. After 24 h of LPS exposure, there is a significant increase in pulmonary vascular permeability, Figure 2B. Surprisingly, XOR inhibition with febuxostat had no effect on lung injury after LPS exposure, Figure 2B.

In order to identify potential mechanisms of lung injury despite XOR inhibition, we first investigated for evidence of apoptosis. We have previously shown that IV LPS leads to significant endothelial apoptosis (Damarla et al. 2014; Singer et al. 2016). As shown in Figure 2C, there is significant activation of caspase 3, as shown by increased cleavage of caspase 3, which was not attenuated by treatment with febuxostat.

Given our hypothesis of attenuating ROS as the mechanism of protection, we tested for markers of oxidative damage. We have previously shown that febuxostat can prevent cigarette smoke‐induced oxidative damage (Kim et al. 2013). Oxidative damage resulting from reactive oxygen species plays a key pathogenic role in organ dysfunction in many disease states including sepsis (Boueiz et al. 2008; Fink 2002). Double‐stranded DNA breaks, as induced by oxidant free radicals (Dedon and Goldberg 1992), lead to a rapid phosphorylation of the histone H2AX at serine 139, which is identified by probing with antibodies against ɣ‐H2AX (Rogakou et al. 1998). In response to systemic LPS, there is marked increase in double stranded DNA breaks, as evidenced by ɣ‐H2AX expression, in lung tissue, Figure 2D. Interestingly, inhibition of XOR does not completely abrogate LPS‐induced ɣ‐H2AX expression. Since XOR activity was diminished to below control levels (Fig. 2A) with febuxostat treatment, we searched for other potential sources of ROS. As LPS leads to an influx of inflammatory cells, that is, neutrophils (Zhu et al. 2017), we measured myeloperoxidase activity as marker of neutrophil‐generated ROS. As shown in Figure 2 Xanthine Oxidoreductase, there is marked increase in myeloperoxidase activity after LPS exposure. There is no attenuation of myeloperoxidase activity in the setting of XOR inhibition with febuxostat, Figure 2E, suggesting that neutrophil‐induced ROS may lead to endothelial apoptosis and resultant pulmonary vascular permeability.

### Inhibiting XOR with febuxostat promotes resolution of lipopolysaccharide‐induced lung injury

In non‐neutrophil predominant states, XOR‐derived ROS have been implicated in endothelial dysfunction (Battelli et al. 2016; Landmesser et al. 2007). Further, in diseased states circulating XOR can bind to vascular beds and lead to endothelial dysfunction. Since our data and previous observations suggest that the pulmonary endothelium rather than the epithelium is a direct target of IV LPS, we reasoned that sustained specific XOR inhibition with febuxostat could lead to improved recovery from LPS‐induced endothelial barrier dysfunction.

**Figure 3:**
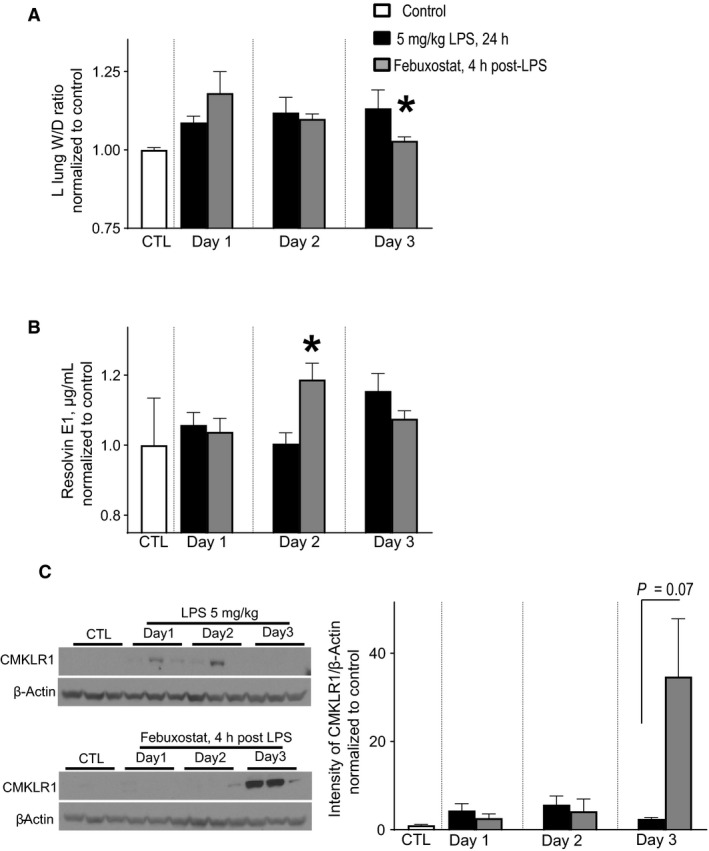
Inhibiting XOR with febuxostat promotes LPS‐induced pulmonary endothelial barrier recovery. Mice were exposed to a sublethal dose of LPS, 5 mg/kg, and a subset were therapeutically treated with febuxostat and after indicated time points lungs were harvested for analyses. (A) Pulmonary endothelial barrier function as measured by lung‐wet weight to dry weight ratios increased significantly in all mice after exposure to LPS. Febuxostat treatment led to a restitution of the pulmonary endothelial barrier by day 3, as compared to LPS exposure alone. (B) Lung tissue resolvin E1 was measured by ELISA. Febuxostat treatment led to significantly higher resolvin E1 levels on day 2 after LPS exposure, as compared to LPS exposure alone. C. Lung tissue chemokine like receptor 1 (CMKLR1) was measured by immunoblotting. Febuxostat treatment led to marked increase in CMKLR1 expression on day 3 after LPS exposure, as compared to LPS exposure alone (Left Panels). Quantification is provided in the Right Panel. *N* = 3–8 mice per group. *, *P *>* *0.05 versus Control.

We therefore focused the next set of experiments on endothelial barrier recovery, that is, lung injury resolution, in our murine model of endotoxemia‐induced lung injury. Given the mortality with 9.5 mg/kg of IV LPS (Fig. 1A), we performed resolution of lung injury experiments using a sublethal dose of LPS, 5 mg/kg, to avoid survival bias. As shown in Figure 3A, mice exposed to 5 mg/kg of IV LPS still had pulmonary endothelial barrier dysfunction on day 3, as evidenced by left lung wet to dry weight ratios, which was significantly improved by treatment with febuxostat after LPS exposure, Figure 3A.

With enhanced resolution of endothelial barrier permeability, we next investigated the role of a proresolution factor. Previous reports have implicated resolvin E1 (RvE1) in resolution of lung injury (El Kebir et al. 2012). Therefore, we measured lung tissue RvE1 over the time course of lung injury resolution. As noted in Figure 3B, there was a significant increase in RvE1 in mice treated with febuxostat, as compared to LPS alone, on day 2 of injury, that is 1 day prior to endothelial barrier recovery (Figure 3A). There are multiple putative mechanisms of action for RvE1; interestingly, the main cells targeted by RvE1 are immune cells and platelets (Fredman and Serhan 2011). In addition, recent reports have identified one of RvE1 main receptor targets as chemokine‐like receptor 1 (CMKLR1) which is expressed on endothelial cells (Kaur et al. 2010). Therefore, we measured lung tissue expression of CMKLR1 over the time course of lung injury resolution. As noted in Figure 3C, there was a significant increase in CMKLR1 expression in mice treated with febuxostat, as compared to LPS alone, on day 3 of injury, which coincides with endothelial barrier recovery (Fig. 3A).

### Inhibiting XOR with febuxostat improves lipopolysaccharide‐induced mortality

**Figure 4:**
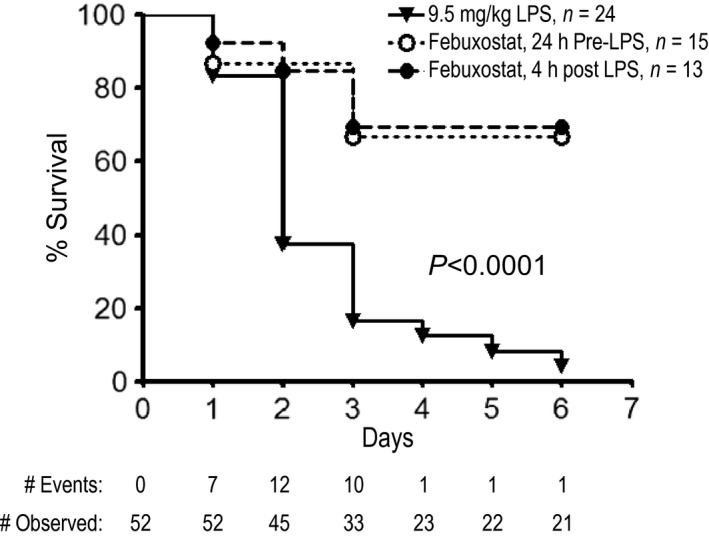
Febuxostat inhibits LPS‐induced mortality. Mice were exposed to 9.5 mg/kg LPS and survival was assessed. A subset of mice were either pretreated with febuxostat or therapeutically treated with febuxostat 4 h after LPS administration. *N* = 13–24 mice per group. The LPS group has a significantly lower survival by Log Rank test, *P *<* *0.0001 versus all others.

Sustained endothelial barrier dysfunction/disruption is a key pathogenic feature leading to sepsis‐induced mortality (Angus and van der Poll 2013; Goldenberg et al. 2011). Having demonstrated the effects of febuxostat on accelerating pulmonary endothelial barrier recovery, we investigated the effects of febuxostat treatment on LPS‐induced mortality. As shown in Figure 4, LPS exposure leads to significant mortality with death starting to occur essentially at day 2. In contrast, mice treated with febuxostat (either pretreated or after LPS exposure) exhibit a significant survival advantage with no significant differences between the treatment strategies.

## Discussion

This study demonstrates that in a murine model of intravenous LPS‐induced lung injury, inhibiting XOR does not prevent/treat acute lung injury. However, inhibiting XOR with febuxostat leads to a more rapid resolution from lung injury, potentially occurring via resolvin E1. Furthermore, inhibition of XOR with pretreatment or therapeutic dosing of febuxostat significantly abrogates sepsis‐induced mortality. To the best of our knowledge, this is the first description of a beneficial effect of therapeutic XOR inhibition with febuxostat in a murine model of endotoxemia‐induced mortality.

XOR is a highly conserved enzyme with activity present in all organs tested, with liver and intestines showing the highest amount of activity (Harrison 2004). Concentration and activity of circulating XOR is low at baseline, however, in response to inflammatory stimuli both can increase dramatically and initiate oxidative damage in organs with low intrinsic XOR content (Berry and Hare 2004; Boueiz et al. 2008; Harrison 2004). This likely occurs via binding of circulating XOR to the vascular endothelium where it can then be internalized (Harrison 2004; Houston et al. 1999; Malik et al. 2011). Due to its ability to bind and act on vascular endothelium of various organs, XOR‐derived oxidative damage is thought to play a pathogenic role in many cardiovascular diseases (Berry and Hare 2004; Boueiz et al. 2008). However, several clinical trials of XOR inhibition in various disease states have yielded mixed results (Dawson et al. 2009a,b; Dogan et al. 2011; Hare et al. 2008). One potential reason for the lack of efficacy may be related to the use of allopurinol or its active metabolite oxypurinol. Allopurinol prevents substrate binding to the co‐factor molybdenum. Therefore, enzyme turnover resulting in ROS formation occurs before effective inhibition of XOR by allopurinol is achieved (Harrison 2004; Malik et al. 2011). Additionally, recent reports suggest that allopurinol is far less effective at inhibiting vascular endothelial bound XOR (Malik et al. 2011). Furthermore, allopurinol being a purine analog may have nonspecific effects on other enzymes of the purine and pyrimidine metabolism pathways (Komoriya et al. 2004; Takano et al. 2005).

In contrast, febuxostat is a highly selective nonpurine analog inhibitor of XOR that binds to the substrate binding site to prevent catalytic activity (Bruce 2006; Ernst and Fravel 2009; Schumacher 2005; Yu 2007), and more effectively inhibits endothelium‐bound XOR thereby preventing vascular inflammation (Becker et al. 2005; Malik et al. 2011). Our data clearly shows that febuxostat effectively inhibits XOR but not MPO activity in lung tissue after LPS exposure, Figures 2A and E. While there is a decrease in LPS‐induced oxidant injury with febuxostat treatment, this is not statistically significant, Figure 2D, suggesting that LPS‐induced lung injury is likely the result of multiple oxidant generating enzymes.

Resolution from lung injury is an active process mediated by several physiological mechanisms (Fredman and Serhan 2011; Robb et al. 2016). Resolvins are a class lipid mediators derived from poly‐unsaturated fatty acids that actively suppress inflammation (El Kebir et al. 2012; Fredman and Serhan 2011; Robb et al. 2016; Seki et al. 2009). RvE1 reduces neutrophil transmigration and can promote clearance of neutrophils (Seki et al. 2009; Yoshimura and Oppenheim 2011). Recent reports have also demonstrated improved survival after intraperitoneal *E. coli* challenge with RvE1 (El Kebir et al. 2012). There are multiple putative mechanisms of action for RvE1; interestingly, the main cellular compartments targeted by RvE1 are immune cells and platelets (Fredman and Serhan 2011). However, recent reports have identified chemokine‐like receptor 1 (CMKLR1) as a main receptor target of RvE1 expressed on endothelial cells (Kaur et al. 2010). Once activated, CMKLR1 initiates prosurvival, proliferative and promigration signaling cascades (Manning and Cantley 2007; Yoshimura and Oppenheim 2011; Zhou et al. 2000). This is particularly relevant after apoptotic‐endothelial injury (e.g., with LPS), as restoration of barrier function requires endothelial cell proliferation and/or migration (Kawasaki et al. 2015; Toya and Malik 2012; Zhao et al. 2006). Our data clearly show marked endothelial barrier disruption after LPS exposure and restoration of endothelial barrier function on day 3 with febuxostat treatment, as compared with LPS alone, Figure 3A. Although the exact mechanism(s) by which XOR inhibition with febuxostat promotes resolution of the endothelial barrier remain uncertain, our data suggests that RvE1‐mediated recovery may be one of them, Figure 3, and is a current focus of on‐going studies in our laboratory.

We recognize the limitations of an IV LPS‐induced sepsis model in completely mimicking human sepsis. However, in order to test the therapeutic benefit of XOR inhibition with febuxostat we deliberately chose an approach where confounding factors of pathogen specificity (e.g., gram positive or negative bacteria) and variability in infection seen in other models, that is,. cecal ligation and puncture or bacteremia, would be avoided. IV LPS administration is a well‐characterized model (Bannerman and Goldblum 2003; Tasaka et al. 2005; Xu et al. 1994) that mimics gram‐negative bacteremia, the most common type of isolated pathogen leading to sepsis (Angus and van der Poll 2013; Mayr et al. 2014). Furthermore, there is a reliable, reproducible, and quantifiable level of lung injury as well as mortality observed, which makes this model ideally suited to test the efficacy of therapies on these parameters.

Finally, a major strength of our study lies in the use of a treatment dosing strategy for febuxostat. Many inhibitor studies show attenuation of injury or mortality with pretreatment. Although, this strategy is critical in identifying pathogenic mechanisms involved in development of injury, pretreatment is difficult to translate to a clinical setting where risk prediction, that is, the potential for a patient to develop sepsis, is less reliable. In contrast, we used a treatment strategy that tests the role of XOR inhibition with febuxostat after initiation of injury. Our data clearly show that treatment dosing with febuxostat is equivalent to pretreatment in preventing sepsis‐induced mortality.

In summary, this study provides compelling evidence that in a murine LPS‐induced sepsis model there is significant XOR activation, oxidative damage, organ dysfunction and mortality, similar to the human condition. Inhibition of XOR with febuxostat, hours after LPS exposure, promotes recovery of the pulmonary endothelium and prevents death. Furthermore, this preclinical study suggests that febuxostat may be a viable therapeutic option in patients with sepsis that needs to be further explored.

## Conflict of Interest

None declared.
